# Foreign

**Published:** 1840-07-18

**Authors:** 


### FOREIGN.

### ANDRAL'S LECTURES ON THE ALTERATIONS OF THE BLOOD.—NO. III.

*Microscopic Researches on the Blood.*

On this subject a good deal of doubt still exists, as the facts discovered are frequently in opposition with each other. In 1673, Lewenhoeck described the blood as exhibiting a number of minute bodies, gifted with motion: he also described the shape and coverings of the globules. Boerhaave's system of *error loci* was based upon the relative proportions of the capillary vessels and globules. Huxham assigned great importance to these globules, and described, from his imagination, the changes that they are subject to. It has since been demonstrated by the microscope, that the globules may be altered as to their form, colour, volume, number, their affinities to each other, and their movements.

*Form*, which is lenticular in the mamifera, may undergo certain physiological and pathological changes. Their form becomes altered immediately after the blood has escaped, and has ceased to circulate; and the same effect is produced by the contact of certain substances, such as the hydro-sulphuric acid. In birds, reptiles, and fishes, their form is elliptical; and some authors assert that sometimes globules of this form are to be met with in man; but, as in these cases the globules had been first dried and afterwards dissolved or diluted, I do not credit the assertion. Their form is changed when they circulate through tortuous vessels, as also when they pass through the very minute capillaries. So many alterations in their forms being thus produced physiologically, must render those which depend upon pathological causes very difficult to be ascertained. In cholera they appeared torn, their surfaces faded, and did not present the circular form. Their appearances in typhus fever are still a matter of doubt. Doctor Donné says their form is altered in dropsy.

*Colour.*—Their colour in the healthy blood varies with the light by which they are seen, as it is a reflected or refracted one. Donné describes globules of a white colour, and of a larger size than the red ones, as existing in the healthy blood, varying in number, but which were found most numerous in cases of dropsy. The red globules in the healthy condition do not always present the same shades of colour. Haller describes them as of paler colour in ill-fed animals; and Donné says they are similarly changed in chlorosis, becoming pale and transparent.

*Volume.*—Their natural size is still uncertain, (120th of a millimètre,) the same globule being at one time larger, at another time smaller.

*Number,*—Is variable, but always considerable, and depends, in some measure, on the quality of the blood: they are more numerous in the arterial than the venous blood.

*Age.*—Denys asserts that, in the fœtus, up to the third week after birth, the globules are found in the greatest number; that they diminish from this period to the fifth month, when they begin to augment to the fortieth year; and from this age to the seventieth year they again diminish.

*Sex.*—They are most numerous in man, whose blood contains 132 parts of globules, out of 1000 parts, that of the woman containing only 92. In those of a sanguineous temperament they are found more numerous, being in man in the proportion of 136 to 1000 of the blood, and in woman, 126. In those of a lymphatic temperament their number is diminished. Nourishment influences greatly the number of globules (Donné;) difference in diet diminishing their number from 132 to 87. They are more numerous in the carnivora than in the herbivora.

*Average proportions of the Globules in 1000 parts of healthy blood.*

| | Average. | | |
|---|---|---|---|
| Dumas and Prevost | 129 | 148 maximum | quantity. |
| | | 115 minimum | do. |
| Lecanu - - | 132 | 148 maximum | do. |
| | | 115 minimum | do. |
| Denys - - | 123 | 173 maximum | do. |
| | | 164 minimum | do. |

In chlorosis, Lecanu rates their quantity at 55 in 1000 parts. I have no reason for supposing that in inflammation their number is increased. In the commencement of scarlatina, Lecanu has found in one case, the number 144; in another 146. The same author has found, in organic diseases of the heart, the number diminish as follows: 101, 79, 51, 45, 43, 41, 40, showing the proportion existing between the globules and the mass of the blood in eight cases of this affection. In diabetes their proportion was 132; and in typhus fever 115. In the healthy state, the globules are separated from each other; but in other cases they show a tendency to coalesce together, so as not to be distinguishable: they move rapidly and regularly. In the normal state, there are vessels which do not carry the red globules, and an obstruction is produced if they by accident become engaged in them.

*Alterations in the Composition of the Blood.*

At present we do not know how the blood is formed. When I was studying chemistry, the explanation that was given of its composition seemed quite clear and satisfactory to all the eminent men of that day; but now every thing belonging to it seems in a state of confusion; but we hope soon to see the uncertainty cleared up.

*Alterations of the normal principles of the blood:*

First class.—First order. Organic matters, fibrine, albumen, colouring matter, fatty matter.

Second order.—Inorganic matters—gas water of the blood, salts, iron.

In a second class we shall treat of those matters which are sometimes found in the blood, but do not enter into its healthy constitution; and in this class we shall find the matters of secretion, about which there is great discussion; we shall afterwards speak of the morbid productions, entozoa, tuberculous matter, &c., which are not found in the healthy fluid, and those substances which are not discovered by the aid of chemistry.

Berzelius states that the difference between the organic principles of the blood, viz., fibrine, albumen, and the colouring matter, is very difficult to detect; and he is of opinion that they may be varieties of one and the same principle or substance.

*Alterations of the fibrine.*—Its natural quantity is unknown, authors differing much on this subject. Davy says it increases in inflammation, and is found to be diminished in scurvy.

Its quantity is said to be altered in typhus. Magendie, by the abstraction of the fibrine from the blood, has induced consequences similar to those of typhus. In plethora it has also been said to be augmented.

*Alteration in its quality.*—When it loses the faculty of spontaneous coagulation, the disease, which we have described under the name of dissolution of the blood, has been produced. The fibrine appears to constitute the organizable matter of the blood, and to form the false membranes when it separates from that fluid.

*Alterations of the albumen.*—It has been asserted, but not proved, that like the fibrine, it is augmented in inflammation, and diminished in scurvy and typhus.

What is the state of the albumen of the blood in dropsy? Is it altered or augmented?

In certain forms of this disease, in which the urine contains albumen, its presence in it has been attributed to its being in excess in the blood. Another theory supposes the kidney to be diseased, and that it secretes the urine charged with it. In support of the first theory, it has been urged that if we inject serum into the blood of an animal, its urine will contain albumen; and also that in many cases it is found in the urine, the kidneys being sound. The result of my observations is, that in the great majority of cases in which there is albuminous urine, the kidneys are also affected. The theory of Graves is, that the granulations which are found in the kidney are formed by the albumen which is stopped in the cortical substance of that organ; but I cannot yield to this opinion, for there are a great number of diseases of the kidney in which they are not granulated, and in which the urine is altered: my opinion is, that the kidney, like every other organ in a state of disease, is no longer capable of forming its secretions, or that it allows the blood to pass through it.

It often happens that Bright's disease begins with hæmaturia, which, after a time, ceases, the kidney preventing the blood from passing through, but allowing the albumen to do so; the office of the kidney permitting it to let pass a number of foreign principles, it may be supposed that when diseased, it will allow of the passage of the albumen. I think that this albuminous urine may be found whenever the kidney is greatly diseased, for I have seen it when this organ was in a state of atrophy or affected with hyperemia, tubercles, calculi, cancer, &c. The blood being impoverished by the diminution of its albumen, may be the cause of certain dropsies.

The albumen may become altered in its qualities, but such cases are hardly known. Berzelius mentions instances in which it has been changed into a fatty matter.

The red colouring matter of the blood may be augmented or diminished with the richness or poverty of the blood. Blue and yellowish colouring matters have been described as existing in the blood: certain principles of the bile are found in the blood in particular affections of the liver. Chevereuil insists upon the presence in the blood of a colouring matter, distinct from a red one; and he describes it as being of a yellow colour, and small in quantity, but capable of being increased. After severe accidents and great operations, the skin of the patient presents a yellow colour, not connected with the presence of bile; and the skin of persons bit by certain venomous serpents, presents the same appearance. From their statements it would appear that there exists in the blood a colouring matter which becomes developed in certain pathological cases, and stains the skin yellow independent of the bile.

The yellow colour of the skin may be produced in three ways.

1. The bile, circulating with the blood, may cause it.
2. The development in the blood of an abnormal colouring principle, which has either been formed in it, or is merely an alteration of a yellow principle existing in the normal blood.
3. Extravasation of blood under the skin.

*Alterations of the inorganic principles which enter into the natural composition of the blood.*—These are, water, alkali of the blood, iron, and salts. The blood contains a gas, which is the carbonic acid, and whose existence in the healthy blood is admitted by the greater number of chemists.

*Alterations of the water of the blood.*—What is the natural proportion of it? In a great number of diseases its quantity is much increased. The serum of the blood contains a prodigious quantity of water: according to Denys, 100 parts of blood contain 78 of water, on the average. Bostock rates the proportion of water at 88; Berzelius at 90. Haller says, it varies from 73 to 93. In the batrachians, Berton found the proportion 90, and in the mammifera and birds, 82 or 83.

When the water of the blood is increased in quantity, the clot formed is small, and the serum is abundant, as seen in anemia, in a high degree, and chlorosis. This condition may be named hydrohemia. Can we augment the quantity of water in the blood by modifying its composition, and by the injection of water into the stomach or veins? The blood disembarrasses itself very speedily of the water which is placed in contact with it. When it does not contain its natural proportion of water, it may take some up to supply the deficiency; but when it possesses its natural quantity, it will not take into its circulation the water injected into the stomach. The experiments of injecting it into the veins have produced nervous affections, coma, convulsions, difficulty of respiration, suffocation, great discharges of water, watery effusions, the brain being found infiltrated, and the lungs œdematous, showing that the water which has been injected separates from the blood, and is thrown out from the torrent of the circulation, producing infiltrations and dropsies. These dropsies may disappear spontaneously by means of purging, copious sweatings, or abundant secretion of urine. After these sudden disparitions of dropsy, not preceded by sweating or secretion of urine, the brain becomes oppressed, coma and death taking place, or the patients may die with a panting respiration, followed by asphyxia, and in this last case the lungs are found œdematous. In these cases the blood has taken into the circulation the fluid of the dropsy, and has thrown it on the brain or lungs. A case is related by Lecanu, of a man who, during forty days, made use of a large quantity of drink. The blood examined at the end of this period contained more water, the globules having much diminished; their proportion at the beginning being 154, and at the end of this time, being but 111. This patient was not restricted as to food, and neither the albumen nor the salts were altered. Great losses of blood very rapidly produce an increase in the quantity of water. A cat having been bled twice within the space of five minutes, the blood of the second operation contained more water than that of the first, and the same results have been obtained by the same means employed on man. Abstinence will cause the same increase, as will also chlorosis. In this latter affection, Lecanu found in 1000 parts of blood, 862 of water. This augmentation in the water may be acute or chronic. When a great proportion of water exists in the composition of the blood, it causes various functional disorders.

When the blood is in a state of liquefaction, serous effusions take place; when it is in a state of dissolution, and has lost the power of coagulating, hæmorrhages are produced; by abundant bleeding we diminish the principles of the blood, thereby rendering it more liquid or fluid. We may bleed an adult very freely, without causing dropsy to take place, but it may be easily induced by large bleedings in children, as we see sometimes after the loss of blood from leeches. Unwholesome food, a long time used, may bring on dropsy, such as a vegetable diet, or substances difficult to digest; and in this instance there are foreign particles introduced into the blood, which may produce its dissolution and bring on scurvy. Can dropsy be produced by living in a humid atmosphere? Flocks of sheep, which have been exposed night and day to great humidity, are afflicted with dropsies and hydatids. I have seen persons who, on coming out of a bath, had a slight œdema of the legs, and a slight puffiness about the face; in these cases the transpiration through the skin had ceased, and the effusion into the subcutaneous cellular membrane had, in consequence, increased. In those countries where cold succeeds rapidly to heat—in regions placed beneath the equator, where a difference of 20 degrees of temperature is often felt between the heat of the day and the cold of the night—sudden dropsies are often produced between the evening and the following morning. In persons sleeping in those countries, on the damp earth, when the night is cold, the cutaneous perspiration ceases, this function is checked, the pulmonary perspiration diminishes, and the blood, in consequence of the suppression of these secretions containing more water, the serous and mucous membranes become vicarious to the suppressed functions. In this way we can also account for the rapid diminution of dropsies, which is sometimes effected by an increased secretion from the skin and kidneys.

The quantity of water in the blood may diminish, and in these cases thirst is often severely felt.

*Alterations of the inorganic principles which naturally exist in the blood.*

Alkali is either free or combined with the albumen or carbonic acid. It is owing to the presence of soda that the blood is always alkaline. I have never found it acid, even in the cholera. Carbonic acid, injected into the blood, will give it an acid character.

Does the iron of the blood diminish in certain diseases? It has been supposed to do so in chlorosis, and the paleness which exists in this disease seems to sanction this suspicion. We are led also to this belief by induction, for there seems to be a relation between the colouring matter and the iron; and if in chlorosis we find the colouring matter diminished, it is a reason for supposing a corresponding diminution of the iron. Preparations containing iron improve the condition of the blood in chlorosis; but if the blood contain its normal proportion of iron, it will not receive any more from the exhibition of medicines containing this metal. Lecanu examined the blood of chlorotic persons; his examination tells us nothing. Dr. Fedish says it contains less iron; we do not place confidence in his analysis. We are therefore uninformed whether the blood in chlorosis contains less iron.

*Salts of the Blood.*—Stevens and Denys insist upon the importance of these substances. The salts and the chloruret of sodium hold in dissolution the albumen and the other principles of the blood, and if the chloride diminish, it will influence the condition of these principles. The addition of the chloride of soda liquefies the blood; the diminution of these salts ought to produce its coagulation, their increase dissolves it. These experiments have been made out of the body.

*Alterations of the composition of the blood by the presence of matters which are not ordinarily found in it.*

1. *Matter of the secretions.*—They do not exist in the normal state, but are found in the abnormal one. If an important secretion be suppressed, owing either to a disease of the secreting organ, or to its being taken away, the matters of such secretion are found in the blood.

2. They may be present in the blood, the excretory ducts being interrupted by some obstacle.

3. A secretion may be interrupted by nervous influence, and its matters will be found in the blood.

4. On account of a superabundant secretion, uric acid, &c., are found in several tissues, without disease of the kidney existing, this organ being insufficient for separating this principle which exists in excess in the blood.

Fatty matter has been found by Christison in the blood, in cases of acute articular rheumatism, and it has been said to exist also in it in cases of diabetes, and in disease of the liver. Tiedemann describes having found the salivary matter in the blood.

*Matter of the Bile.*—Some cases exist, in which the colouring matter of the bile has been found in the blood. In jaundice, the blood is turned green by the addition of nitric acid, and the matters of the bile have been found in most of the solids and fluids. Is jaundice the only disease in which the blood has been found im pregnated with the matter of the bile? In those affections which are called biliary, Martin Solon says he has found the bile in the blood; I have tried to find it in the variety of biliary pneumonia, but I have never succeeded in detecting the matter of the bile in the blood, in any disease but icterus.

*Matters of the Urine.*—Uric acid has never been detected in the blood. The blood has never been found acid, and if it ever exists in it, it must be combined with a base. Urea has been detected in the blood. Dumas has removed the kidneys in living animals, and has found in their blood urea, which is the fundamental principal of urine, and which cannot be formed without its presence. Tying the ureters produces the same results. We shall now see what the cliniques teach us. When the kidneys were diseased, we have seen the blood passing through the filter of these organs, and mixing with the urine; at other times the albumen alone was allowed to pass through. What becomes of the urea in these cases? Sometimes the urine is found deficient in it, although albuminous; at other times it has been found in it. In some cases of albuminous urine, the blood presented nothing particular; but in other cases I have detected alterations in its compotion, as it contained a quantity of urea. Cases are related in which the solids contain urea; others, in which the urine has been met with in the principal secretions: I doubt them. Fisher mentions cases where it was secreted by a great number of organs. Is the presence of urea in the blood followed by any bad results? If the secretion has been slow, the accidents are not very evident; but if it has been caused rapidly, by taking away the kidney producing a suppression of urine, the results are very evident. In cases where an acute inflammation has suppressed the secretion of urine, typhoid symptoms have followed, with all the symptoms of nervous re-action. Milk, or its principle, caseum, has been said to have been found in the blood; but these facts require confirmation, as chyle, albumen, or fatty matters, might have been mistaken for it. I do not know a well-authenticated instance in which its presence has been ascertained in the blood. In puerperal fevers, the caseum of the milk may perhaps be found in it.

*The products of morbid secretions may be found in the blood.*

*Pus.*—When this morbid production has been detected in the blood, we must inquire into its formation. It has either been formed in the blood itself, in consequence of this fluid having been converted into the pus, or it may have been secreted from the sides of the vessels in the situation in which it has been found. If thus secreted by the walls of the vessels, it may be found, distant from the spot where it was formed, as is sometimes seen in inflammation of the veins of the arm, whence it is carried with the blood into distant parts. Lastly, the pus may have passed into the blood by absorption.

In the following acute diseases, pus has been found in the blood: in acute metritis, phlebitis, (general or local,) metroperitonitis, and once in acute articular rheumatism. In chronic disease it has been found in phthisis. In what part of the vascular system, excepting the lymphatics, has pus been found? It has been seen in the heart mixed with blood, and frequently in the veins, but I have never found it in the arteries, except in one case of circumscribed arteritis, produced by a partial inflammation. What are the pathological circumstances which accompany this purulent condition of the blood? They are numerous; sometimes large foyers of suppuration, or large suppurating surfaces; suppurating wounds and phlebitis; at other times there exist none of these circumstances, and we merely find an alteration of the blood, which is itself the source from whence the pus is produced. In other cases it is found disseminated and infiltrated through the blood, and closely intermixed with it. It may also be met with in drops, floating in the blood, but distinct from it, or forming small depôts or abscesses, enclosed in a false membrane, which separates it from the clot of blood in which such a purulent deposit may be found enclosed. These drops of pus have been most frequently met with in the veins; whereas we more frequently find in the heart deposits of pus, imbedded in clots of blood but distinct from them.

Donné says, that pus, added to the blood in a coagulated state, produces its liquefaction. According to Maude, blood added to pus coagulates it. The same author, in beating up blood, perceived a membrane forming itself around the rod which he employed. If he added pus to the blood before he beat it up, this membrane did not form; however, if the quantity of pus added were small, the membrane appeared, but in a very indistinct form, and if the quantity of pus were increased, there was none formed.

If we add pus to blood deprived of its fibrine, and examine it with the microscope, we perceive the coats of the globules becoming infiltrated, the globules themselves turning opaque, their forms lengthening, and, finally, they disappear.

From the fact of the globules of the blood differing from those of pus, it might be inferred that no difficulty would be found in distinguishing them from one another: such, however, is not the case. The globules of the blood vary in aspect, colour, &c. By the action of ammonia, pus becomes congealed; and on the addition of blood, the globules are dissolved. In some cases, however, certain portions of the blood become congealed by the ammonia; and this mode of experimenting is therefore inconclusive, and the *experimentum crucis* is yet to be discovered.

How does pus find its way into the blood? It must either be absorbed or secreted by the walls of the blood-vessels or of the heart, or formed by the blood itself.

The absorption of pus is a very rare case, and I do not think that its presence can be attributed to such an action; for if such were the case we should meet with it more frequently in cases of phthisis, for example; and even if it did thus find its way into the blood, it may undergo some alteration. I am, therefore, of opinion, that, in the majority of cases in which pus is found in the blood, it is not owing to absorption.

Pus is often found in the blood after phlebitis, but this disease will not explain all the cases in which it is so found. In some instances it has been detected where no traces of phlebitis existed.

In some cases, where pus is found in a vein, it mixes with, and is carried along with the blood; but, in the great majority, clots are formed, which imprison the pus, and prevent it from circulating with that fluid.

Jessier mentions, that pus secreted in a vein can never enter into the torrent of the circulation, as it becomes isolated in the place where it was formed. I am not so exclusive, for I have seen cases where this plug or clot was not formed, and where the pus was carried into the circulation. Gendrin is of opinion, that in certain cases the blood becomes altered, and is changed into pus. This theory comes from Dehaën. I am inclined to this view, which explains certain morbid phenomena.

Duplay relates a case in which the greater number of the blood-vessels contained no blood, but were filled with pus; the patient presenting all the symptoms of a purulent fever, unaccompanied with any signs of phlebetis.

To explain those cases in which we find abscesses or purulent deposits in different places, either in acute or chronic forms, we must suppose that the pus first becomes mixed with the blood, and afterwards separated, but by what process is uncertain. I think it probable that, in these cases, the blood itself is in fault, and that it forms the pus as it may form urea, under different circumstances.

The theories to explain those purulent deposites are therefore uncertain, and they have been ably discussed by Dehaën.

*Encephaloid matter in the Blood.*

Has its presence been ascertained? The blood may present an alteration which may render it a very difficult question to distinguish it from the encephaloid matter.

In the cancerous diathesis we sometimes meet semi-coagulated blood, strongly resembling this encephaloid matter, plentifully deposited in the greater part of the solids and in many of the veins, and more especially in the vessels in the neighborhood of the cancerous ulcer or deposit. It has been in particular in subjects who presented such encephaloid deposits in their solids, that this matter has been found in the blood, and which so closely resembles it; but I will not affirm that it is the encephaloid matter itself.

I do not know whether tuberculous matter has been detected in the blood.

An Italian has found, as he supposes, entozoa existing in the blood. It has been said that the presence of hydro-sulphate of ammonia, calculi, &c., has been detected in the blood.

*Principles in the blood inappreciable by the help of chemistry or of our senses, but rendered evident by those organs becoming affected to which the blood is sent; this fluid presenting no visible alteration.*

The blood of an animal exhausted by fatigue undergoes some alteration; for if injected into another animal, it will produce death, accompanied with symptoms of poisoning. If the blood of an animal affected with anthrax, (maladie charbonneuse) be injected into another animal, this latter will be infected with the same disease. We have, therefore, reason to suspect some alteration of this blood, although it will be impossible for us to detect it by any means with which we are acquainted. In the old writers we often meet with the words, "acrimony or sourness of the blood." These words may, perhaps, comprehend more than they seem to express. May they not refer to some alteration of the blood furnishing unhealthy elements or matters of secretion, which, thus deteriorated, would produce diseases in the solids? If we look for the origin of those general formations or deposits of uric acid which we find in the solids, we may find it in the blood which deposits in various parts of the solids, cartilages, bones, &c. By reflecting on these circumstances, and arguing from hypothesis, I think it more probable to refer to the blood those general morbid secretions, as tubercles, than to suppose an alteration of the solids in which we may find them. Do those furuncles which we see disappearing and returning after uncertain intervals, owe their origin to some alteration of the parts of the skin on which they are situated, or are they owing to some particular alteration of the blood? I am inclined to refer them to the latter cause, as we often find other secretions in a morbid condition producing discharges from the mucous surfaces, or disorders of the nervous system; and it is evident that these general symptoms depend upon some common cause. In a great number of diseases which have their origin in a common cause, we may ask if this cause is not to be found in the blood itself.

The blood may become altered by substances coming from without, and mixing with it. The kind of food may modify the blood, as well as different conditions of the atmosphere; many poisons which are capable of being absorbed by it—miasmata, virus, &c.

In the cases just alluded to, is the blood really altered, or does it merely carry along with it the elements of these substances?

In some of them it is not altered, and can disembarrass itself of these poisons; in other instances it becomes really altered, by the contact of these poisons, and the morbid condition produced is owing to the combined influence of the poison, and of the alteration or morbid condition of the blood itself.—*Lon. Med. Gaz.*

---

*On the Formation of Cancer of the Veins, and the possibility of communicating Carcinoma from Men to Animals.** By Dr. B. Langenbeck, of Göttingen.—Late investigations, and especially

* The author by the terms *cancer* and *carcinoma*, appears to mean the whole family of malignant tumours; his cases relate chiefly to the medullary or encephaloid species.

those of Carswell and Cruveilhier, have shown that the occurrence of cancer within the veins is by no means rare; and the latter has found it so frequently, that he has come to the opinion that all cancers are originally developed in the venous capillaries. But, however frequently the veins may be the seat of carcinoma when the uterus is affected with that disease, yet in some cases they are undoubtedly quite healthy, and I therefore cannot coincide with Cruveilhier's opinion. I am rather inclined to regard the frequent occurrence of carcinoma in the veins as in most cases something secondary, like phlebitis, a disease which is also frequently joined with carcinoma, and especially with carcinoma of the uterus.

But, with however little confidence it can be at present held, that the capillary veins are the seat of origin in all cases of primary cancer, they are still very frequently to be regarded as the seat of the development of secondary carcinoma, and I would even hold, that in all cases in which a primary cancer exists, or has existed, and in which secondary cancer appears in some other part of the body, it is constantly developed from the capillary vessels, (I will not say veins.)

The answer to the following questions appeared to me of the highest importance. How does the matter of cancer get into the veins, and how does cancer develope itself within these vessels? One might expect either—1st. That the disease forms as a cancerous degeneration of the walls of the veins, which are, no doubt, subject to the affection like all other tissues. But against this idea is the fact, that in almost all cases of cancer of the veins, the disease is connected with the internal as well as the external surface of the vessel: or 2d, we might conceive that the cancerous matter grows from without inwards into the cavity of the vein; but this could account only for those cases in which the veins immediately adjacent to a cancerous mass are diseased, but not for those in which the veins at a distance are affected; or 3d, it might be, that separated fragments of a cancer pass into the veins destroyed by ulceration, and accumulate at some part of their interior; but in this case it would be difficult to imagine how the masses of cancer within the veins should (as they often evidently do) possess vessels.

The author considering each of these modes of explanation equally unsatisfactory, proceeds as follows. The cancerous matter which I have found in the veins lies in part quite loosely in them, without any connection with their walls, and is in part slightly adherent to their internal surface; and lastly, in part it forms one mass with their walls, which are in like manner converted into carcinomatous tissue. The origin of these differences remained for a long time obscure to my mind; but two cases of incipient carcinoma of the lungs, which were developed secondarily to carcinoma of the uterus, completely explained it. I convinced myself that the development of the cancer of the veins depends on that most remarkable property which the minutest cancerous molecules, the microscopic cancer-cells, possess, of developing themselves into cancerous tumours even when they are completely isolated, and without any organic connection with each other in the circulation.

The development and the growth of carcinoma, as well probably as of all morbid tissues connected with the organism, depends, as is well known, on the growth of simple cells, and takes place according to the same laws which Schleiden first proved in plants, and which Schwann has shown to obtain in the normal animal tissues, and Müller in many morbid structures. The analogy of the mode of growth of all organic structures, which is so clearly proved by the investigations of these authors, is remarkably confirmed by the fact, which I have observed, that the germ-cells of a cancerous tumour, introduced separately into the fluids of the body, or passing accidentally into the circulation, may develope themselves independently, or in any part of the capillary system, into carcinomatous tumours, just as, in the lower plants, any cell separated from the plant may continue to grow independently.

Both the cases from which the author was enabled to draw this conclusion were cancers of the uterus. In one, nearly the whole uterus was destroyed by ulceration, and a recto-vaginal fistula had formed. In the other, only the cervix uteri was destroyed, and it was converted into an ulcer with callous margins. In the bodies of both patients, there were found in the uterine and pelvic veins light yellowish-red granular coagula, (the *matieres cancereuses* of Cruveilhier,) consisting of soft fibrine, coagulum, pus-globules, and small cancer-cells, whose diameter was twice as great as that of the pus-globules. The greater part of the coagulum was formed of very small, rather long, rounded transparent granules, half as large as blood-globules, and in every respect similar to the finely granular matter which one finds in the simple microscopic cancer-cells, so that I could not but regard them as the contents of destroyed cancer-cells set free. The iliac veins, the inferior cava, and the right side of the heart, were full of dark fluid blood; in that in the veins I found with the microscopic granular cells with very distinct yellow-coloured nuclei, and a quantity of the fine granules which formed the principal part of the coagula in the hypogastric veins. In the blood of the right side of the heart I detected, with the naked eye, yellowish-red soft coagula, consisting of the same microscopic elements as the coagula of the pelvic veins. When I opened the pulmonary artery from the right side of the heart, I found in it the same reddish-yellow granular coagula as in those veins, only that here they appeared much firmer, and in the finer branches of the pulmonary artery were here and there distinctly united with the inner surface of the vessels. In the larger branches of the pulmonary artery, these coagula lay completely free, and partly filled the cavity of the vessel; but the finer the divisions of the artery became, the more completely did the vessels seem blocked up, and the more intimate was the union of the coagula with their walls. Under the microscope, these coagula were found to consist almost entirely of large cancer-cells, of which the majority seemed five or six times as large as blood globules, and which were in no respect distinguished by their form from the cells in the tissue of the cancer of the uterus.

These coagula consisting of particles of cancer in the pulmonary artery, admit of a two-fold explanation. Either, 1st, they were merely dead aggregations of separated particles of the cancer of the uterus, which had passed into the circulation through the ulcerated and open uterine veins, and had proceeded with the blood through the right side of the heart into the pulmonary artery, and accumulated in its smaller branches, (but to this view the circumstance is opposed that the uterine and hypogastric veins contained exactly similar coagula, but which were much softer than those in the pulmonary artery, and had nowhere any connection with the walls of the vessels, and were composed of a number of smaller cancer-cells, and only in part of nuclei;) or, 2dly, these coagula were true cancers in the course of development and vital growth, whose form only appeared somewhat modified by the adjacent parts, the walls of the vessels. In favour of this view was the fact of the more complete formation of the cancer-cells in these coagula in the pulmonary artery, and their complete union with part of the walls of the vessels. Besides, when I traced the coagula into the minutest branches of the pulmonary artery, I came upon small flattened roundish cancers at the surface of the lungs, just under the pleura, into which I could trace small branches of the pulmonary artery completely filled with cancerous matter. In the neighbourhood of these incipient cancers, the coagula were so intimately united with the walls of the vessels, that they could not be distinguished from one another, but presented beneath the microscope a homogeneous cancerous tissue. But when the vessels passed into the small cancerous masses, then they completely lost their cylindrical form, by the growth of the cancerous structure through their walls. With the exception of the small cancers, of which there were ten or twelve in each, the tissue of the lungs was perfectly normal, though from the obstruction to the pulmonary circulation very œdematous.

I think it is very probable that, in most cases, cancer of the lungs developes itself within the branches of the pulmonary artery, from molecules of cancer which have passed from some primary cancer into the venous blood. Cancer of some other part of the body almost constantly precedes its development in the lungs; and the cases of primary cancer of the lungs are extremely rare. Bayle never saw but one, and Bouillaud only two; and Andral has never seen a case in which the lungs alone were affected.

That the minutest portions of a cancerous tumour, the cancer-cells, still possess the power of developing themselves independently into cancerous tumours, though separated from their original stock, the primary cancer, and planted on a foreign soil, can scarcely appear strange when we remember that the germ of the ovum, itself nothing but a cell, and, in fact, remarkably like a large cancer-cell, separates after conception from the ovary, to be developed independently in the uterus, or quite away from the maternal body. Like the germ of the ovum, every individual cancer-cell must be regarded as an organism endowed with vital power and capability of development, which, even when separated from all organic connection with its original soil, can yet continue to grow independently, so long as it is in the neighbourhood and under the influence of living organic tissues.

It was of considerable interest for me to determine whether cancer-cells, introduced into the circulation of an individual of a different species, would develope themselves into cancerous tumours in the capillary vessels. The endeavours of Alibert to produce cancer in animals and men by inoculating them with cancerous ichor, were, it is well known, ineffectual; and I repeated the experiment several times, both in dogs and rabbits, in vain. But if my observations on the development of cancerous tumours from simple cancer-cells were correct, it was clear that the seeds of the disease were in them only, and that a communication of cancer could be effected by nothing but them. This being assumed, it was explicable why the experiments of inoculation with the ichor had failed; for in it, as it is commonly taken, no cancerous molecules are ever found. I therefore determined to introduce some cancer-cells from recently extirpated human cancer, into the circulation of animals. Several rabbits, into whose external jugular or saphena veins I introduced fluid from fresh carcinoma, died between twelve and twenty-four hours after, from a remarkable obstruction which the injected fluid had caused in the capillaries of their lungs; for all died with dyspnœa, and all their lungs exhibited a considerable number of small ecchymoses. But, contrary to my expectation, the following experiment on a dog was important in its results:—

I took eight ounces of blood from the femoral artery of a large, strong, two-year old dog, and removed its fibrine by stirring. I then took about half an ounce of a whitish cancerous fluid, which had been scraped from the cut surface of an enormous medullary cancer, just removed from a young man's arm, and carefully purifying it from all the pieces of the tumour that were mixed with it, I mixed it with the defibrinated blood, and injected it into the femoral vein of the same limb. Two days afterwards the dog was ill and feverish, but had no affection of the respiratory organs, and in eighteen days he was quite well. Some time afterwards, he began to grow very thin, and on the 10th of August I killed him by pithing. On opening the chest, the lungs were found apparently healthy; but on the anterior surface of their upper lobes there were two or three clear-bluish, flattened, and rounded tumours, of the size of a lentil, which were remarkably like the small cancers of the human lung described above, and, under the microscope, presented the texture of cancer. In the substance of the middle lobe of the left lung there was a large, hard, circumscribed tumour, of the size of a large field-bean. The pulmonary tissue around it seemed quite healthy; but, on its cut surface, it presented all the appearance of a cancerous tubercle, consisting of a hard, homogeneous, clear-bluish substance, in which there were here and there points of blood, which, under the microscope, looked like convoluted capillaries.

Now, as this tumour possessed a peculiar and definite tissue, and was organized with blood-vessels, it could not possibly be a mere accumulation of the cancerous matter injected into the blood, but it must, if it were of a cancerous nature, have been formed by the growth and continued development of the cancerous molecules. The microscopic examination of the fresh tumour left no doubt whatever of its carcinomatous nature. It consisted of large, clear, juicy fibres, of the thickness of primitive muscular fibres, between which cells of 1-100th of a line in diameter were thickly scattered. In the clear fluid which could be squeezed out of it there were smaller cells, partly of the size of blood globules, and partly only half as large as them; and, besides, it contained a quantity of fat. The same microscopic elements were found in the medullary cancer of the humerus from which the injected fluid was taken, and the similarity in structure of the two tumours could not be doubted when they were compared with each other. The cancer of the lungs in the dog was distinguished from that of the upper arm of the man only by its greater hardness, its larger fibres, and the large, dark, granular cells, with distinct nuclei, which were here and there scattered in it, just as I have so often seen them in scirrhus of internal organs, though never in medullary tumours. The cancer-cells, therefore, appear to be a new development from the injected substance: and hence, I think, they confirm the idea, that the medullary and the hard cancer are only different forms of essentially the same disease, and may pass the one into the other.

In the great frequency of cancerous diseases, it will be an easy matter to repeat and vary the experiments I have instituted, and, without doubt, they will succeed alike with all the varieties of cancer. It is still to be determined, how far the vitality of the cancer-cells is dependent on the life of the organism; and whether cancer-cells, taken from the cancerous tumours of a dead body, can develope themselves into carcinomatous tumours, as well as those from cancerous tumours just removed from the living body, and still warm.—*London Med. Gaz., from Schmidt's Jahrbücher*, Bd. xxv. hft. 1, p. 99.

---

*On Severe Injuries of the Joints, and their Treatment.* By Rutherford Alcock, Esq., K. T. C.—The author commenced by showing, that the only information we possessed on so important and complicated a class of injuries, was to be found scattered in various medical journals, and chiefly in the works of the military surgeons; and that even when all these fragmental data were collected, they were far from furnishing any thing like a complete or comprehensive classification of the various kinds of injury defined in reference to certain fixed principles of treatment. To supply this was the object in view.

In reference to this class of injuries there was not only the grave consideration of amputation, and the necessity of determining in which cases there was a fair prospect of saving a useful limb, but there were other operative means, such as the excision of an articulating end of a diseased or injured bone, by which a limb might be saved without all the hazard of the reparative action, when the end of the bone was seriously implicated. Cases in which this alternative offered, required to be carefully defined.

Many of the general conclusions were founded upon a close analysis of the nature, progress, and results of ninety-six cases of severe injury to the articulations. Such of those conclusions as were numerical he had thrown into a tabular form, to which he adverted under the following heads:

Mortality of these injuries in comparison other kinds, and relative mortality in each articulation.

Comparative frequency of these injuries with those of other parts of the body, and of one articulation.

Causes of mortality in those who died while under treatment for the original injury—those who died after primary or secondary amputation.

From a review of numerical results, under these heads, the author passed on to the consideration of particular cases forming types of classes, and the principles of treatment applicable to each. Many highly interesting cases were shortly narrated, proving essential differences between injuries apparently similar in many circumstances, but yet requiring the application of different principles of treatment.

Upon these data, and the conclusions drawn from them, the author founded his grouping of the injuries into three classes, having reference to certain leading features of treatment.

*Class* 1.—Consisting of lacerated wounds external to the capsule. Incised or lacerated wounds penetrating the capsule. Penetrating wounds with abrasion of articulating surfaces. Simple fractures into joints with more or less displacement and ligamentous adhesions. Fissuring of articulating surfaces from compound fractures, complete or partial, in the vicinity, but without displacement of bone within the capsule. In this class are included those where the great majority of limbs may be saved, and where it should be a principle of practice to attempt it.

*Class* 2.—Foreign bodies lodged in the ends of bones, either not presenting on the articular surface, or on the same level, and smooth.

Foreign bodies traversing the ends of bones, without detaching fragments.

Internal laceration of ligamentous structure—lesion of blood-vessels, with or without temporary displacement of articulating surfaces.

The *second* forms an intermediate range between those in which the principle is laid down that they may be saved, and those in which the contrary rule holds, viz., that the attempt ought not to be made. These, of all the injuries to joints, most require accurate diagnosis and sound judgment in determining the line of practice, whether to attempt to save or at once to condemn. The author had succeeded in saving many of these; but it certainly was not always judicious to make the attempt.

In the kind, "Foreign bodies traversing the ends of bones without detaching fragments," an example was presented after the meeting, and examined by many Fellows of the Society. The author had succeeded in saving a limb so strong, that the man had walked from London to Liverpool. A musket-ball had entered at the inner edge of the patella, fracturing it, and traversing the internal condyle, and came out near the centre of the popliteal space.*

*Class* 3.—Compound fractures into joints with displacement and roughened edges. Foreign bodies projecting into articulations, or traversing with extensive injury to structure. The third class includes those kinds, where the principle of practice is to amputate without delay—the injury being of an irremediable character, the system from the first moment takes alarm, and each succeeding hour rapidly diminishes the powers of the patient. He had known only one case (except of the hand or foot) recover, where there was fracture into the joint, causing displacement and roughened edges, and that was of the elbow.

Many valuable preparations were shown to the Society, exemplifying different kinds of injuries to the articulations, and their effects.

Mr. Charles Hawkins, in reference to a case which had been alluded to by the author, now under the care of Mr. White, in the Westminster Hospital,* mentioned the following:—A lit-

---

* The particulars of this very interesting and almost *unique* case, were the following. The patient was 23 years of age, of fair and florid complexion. In January 1836, he was wounded at the battle of Arlaban. Mr. Alcock happened to be riding past the spot where he fell, a few minutes after the wound was received, and observing that the injury was at the knee, dismounted to ascertain the precise nature and extent of the wound, before any swelling or inflammation should supervene, and render the examination impossible. He passed his finger into the wound, and ascertained that a musket-ball entering at the internal edge of the patella, and partially fracturing that bone, had passed through the internal condyle, emerging close to the inner hamstring near the central horizontal line of the popliteal space; its course from its entrance being nearly obliquely upwards. He could detect no fracture or fissure of the articulating surface; the ball had made a free passage without detaching any fragment of the condyle from the shaft. He determined on endeavouring to save the limb, and had the man conveyed to the rear. Mr. Alcock remarks that, "late at night one of the regimental surgeons—quartered with his men in the same village as myself—requested I would see a wounded man, whose leg he thought should be removed without delay. On arriving at the quarters, I recognised in the patient the man I had already seen in the field. The limb was swelled and so considerably inflamed, that examination was no longer possible, and had I not previously satisfied myself of the nature of the injury, I should probably have taken the same view of the case as my colleague. As soon as I had succeeded in having the wounded conveyed to the hospitals in Vittoria, I took good care of him. The inflammatory accidents were less violent than I had anticipated, and even the suppuration not excessive, and in thirteen weeks he was upon crutches."

He has now fully recovered the use of his leg, and although there is some degree of anchylosis limiting its flexion, he is able to take great exercise, as stated in the abstract above. Since his return to England, he has been once laid up for a short time, by what he describes as an abscess about the knee-joint, with considerable swelling.

This is one of the three cases brought forward by Mr. Alcock, to prove that destruction of the knee-joint was not, necessarily, the result of the passage of a ball, opening the capsule and passing through the spongy end of the femur. Doubtless it was impossible to predict with certainty, what degree of inflammatory action might ensue, and a certain degree of risk must be attendant upon every trial to save a limb thus injured. In the particular class of cases here referred to, Mr. Alcock considers that the chances of success are sufficient to warrant the attempt.—*Reporter*, LANCET.

* This case is one of much interest, and we shall give a detailed account of it at its termination. The patient is a boy about six years of age, who, while riding behind a cab some months since, got his leg tle boy, while riding behind a carriage, got one of his legs entangled in the wheel, and sustained a fracture of the thigh. He was brought to St. George's Hospital, when it was found that he had a large wound in the ham, through which the extremity of the femur protruded. The limb was removed. On examining the knee-joint it was found to be perfect, the bone having been merely torn from its epiphysis. The artery and vein were found to be uninjured.

Mr. Bransby Cooper observed, that the author of the paper seemed to consider that there was much less danger in laying open the synovial membranes of joints, than had been usually supposed, unless, indeed, there were some foreign body in the cavity of the joint keeping up irritation. He (Mr. C.) thought these views correct, and would mention a case in illustration. A man received an injury by which one of the patellæ was carried away entirely, and the joint laid open, the synovial membrane being torn through, and the articulating surfaces of both the tibia and fibula exposed, but not otherwise injured. It was at first thought advisable to amputate the limb immediately; but on consultation with another practitioner, it was determined by the gentlemen in attendance, as the joint was uninjured, except so far as its synovial membrane was concerned, to try to save the limb. The surgeon who was consulted entertained the same views with reference to injuries of this membrane, as those advanced in the paper before the Society. The wound was covered with lint dipped in oil, and a splint placed behind it to keep it quiet. The patient passed a quiet night, and on the following morning was free from irritation of any kind. In short, he perfectly recovered without the occurrence of any bad symptom, and has a very useful joint; he rides on horseback as well as before the accident, and is a good cricketer. The wound in this case healed by granulation, and the cicatrix exhibited several cartilaginous deposits with an evident attempt at the formation of a new patella. This case occurred in the practice of Mr. Edlin, of Cambridge. Entertaining the same views with reference to laying open the synovial membranes as those advanced by Mr. Alcock, he (Mr. C.) trusted that the time was not far distant when it would be found to be good surgery to lay white swellings freely open, and to allow the limb to be restored to health by the secondary operation of nature.

Sir B. Brodie had seen several cases in which the synovial membrane had been wounded, and the joint remained long exposed, yet the patients did well, and the joints were restored to healthy action. In illustration of the correctness of the observation, that laying the synovial membranes freely open was often attended by no bad consequences, he would relate the following case:—An officer in the artillery suffered much pain and inconvenience from what appeared to be a loose cartilage in the knee-joint. At the earnest entreaties of the patient, he (Sir Benjamin) consented to remove it. On cutting into the joint, he was surprised to find a large fleshy tumour, about the size of the two last phalanges of three of his fingers. It was attached by a broad base to the inner surface of the synovial membrane. The limb was kept quiet for two or three days, the incision was then somewhat enlarged, in order that the base of the tumour might be reached with more facility, and the diseased growth removed. The wound healed by the first intention; severe inflammation, however, came on, attended by excessive distension of the joint, the cicatrix gave way, and the bed was literally deluged with synovial fluid. The flow of the synovia continued for three or four weeks, at the end of which time the wound healed, and the patient did well.

In another case in which he removed two loose cartilages from the knee-joint, adhesion took place, and the wound healed—inflammation followed, and the synovia escaped as in the last case, the discharge continuing for about a fortnight. The joint was completely restored to healthy function. Laying open the joint freely in these cases was always advisable, as mischief was likely to result from the collected fluid not having a ready means of exit from the joint. In white swellings there was generally some scrofulous disease of the bones, and he therefore did not anticipate any good results from laying the joints open, as proposed by Mr. Cooper. In children, however, in these cases, making an incision into the articulation, and afterwards supporting the patient's strength with steel medicines, was often successful in bringing the case to a favourable termination.

Mr. B. Cooper had intended to limit his observations to the disease in children.—*Trans. Roy. Med. and Chirurg. Soc.*, April 28, 1840.

*Lancet.*

---

*New mode of treating Scarlet Fever in its more fatal forms. Treatment of Croup.*—At a meeting of the Medical Society of London, April 20, the President, Dr. Clutterbuck, made some observations on the fatality and prevalence of scarlet fever at the present time. He remarked that

---

entangled in one of the wheels. The thigh was fractured. On admission into the hospital a large wound was found in the ham, through which the extremity of the femur protruded, having been torn from its epiphysis. The vessels lay in front, and were uninjured. As the joint did not appear to be involved, Mr. White determined on attempting to save the limb. He accordingly removed the extremity of the femur, and placed the bone in its natural position. There was much constitutional irritation for a long period, with purulent discharge from the injured part. The boy is now, however, recovering his health, and will have a somewhat useful limb.—*Reporter*, LANCET.

the treatment of this affection, as now pursued, in many cases, was to him very unsatisfactory; so much so, indeed, that he sometimes scarcely knew on what principles he was acting. He had rarely seen sufficient to convince him that the mode of treatment pursued had much to do with the cure of the case. He believed, indeed, that the treatment often effected nothing, while it was as frequently injurious. In those cases in which the eruption began to fade, the pulse became irregular and the brain disturbed; stimulants were recommended to be given immediately. He had used them with much freedom, but the result had been any thing but satisfactory to him. It was easy enough to bring up the pulse for a time, by the use of stimulants; and if this were the sole object to be obtained by their use, it might be done. But the production of a more powerful state of the pulse was not always beneficial; on the contrary, the patient seemed sometimes worse after this was effected. The pulse, too, soon flagged again, and its power was not to be renewed by the repetition of stimulants at will, however freely they were employed. He believed that the stimulus of alcohol injured the brain, which was the organ chiefly affected by the injurious operation of the poison of the disease. When there was an equality of the symptoms present, the eruption being sufficiently abundant, and the throat but moderately affected, the case was disposed to end favourably. In bad and fatal cases, however, the eruption was apt to disappear; and soon afterwards, stupor, delirium, a sinking state of the pulse with great exhaustion of the system to succeed. Now, if it were possible to renew the action of the skin without injury to the brain, we should reason, *a priori*, that the brain would be benefited. This was to be effected by counter-irritants, and the more general the counter-irritant the more likely was it to be successful. The opposite effect on the brain was likely to result from the employment of stimulants.

He had lately been attending five members of a family with scarlet fever, four of them being children, and one a female servant. Two of the children died; the other two, with the servant, were now out of danger. All of them had been in a state productive of great alarm. He did not see the first case early, and the child died. He saw the second case sooner, and adopted the plan which had been pursued with the first case, viz., the early administration of stimulants, for the purpose of rousing the system: this was at first effected, but the symptoms of depression returned; the child went from bad to worse and died. The plan of treatment pursued in these cases being so unsuccessful, he felt it difficult to determine how to act with the two other children. It occurred to him, however, that if he could get a return of action to the surface of the body, the disease might be brought into a more favourable form. As the readiest mode of effecting this was by the use of the warm bath, impregnated with mustard, he determined on putting this plan in operation. A warm slipper-bath, that being large enough, was prepared for the third child. Owing to some mistake in the directions, as much as two pounds of powdered mustard were placed in it, and in this mixture the child was immersed. The stimulus of the bath soon became intolerable, and on removing the child at the expiration of three or four minutes, the skin was found to be intensely red; this redness continued, the child rallied, and did well. This case was encouraging. The servant-girl, a strong country wench, twenty-three years of age, became in imminent danger; her pulse was frequent, small, and irregular; so much so, indeed, that one practitioner in attendance suspected this condition of the circulation could only be the result of cardiac disease; he (Dr. Clutterbuck) however, thought the brain to be chiefly at fault, particularly as the girl, previously to the occurrence of the fever had been in good health. In addition to this condition of the circulation, the surface was pale and cold, and there was great depression. The mustard bath was used, the action of the skin was restored, sweating was induced, and the patient recovered. In the case of the fourth child, the same remedy was employed with the same results. Looking at the disease as presented to us in cases of this description, the remedy employed appeared to be a rational one, as tending to bring the patient into that favourable state which is present when the symptoms of the disease are more generally diffused. The mustard-bath was a remedy which he should never think of using, except for the purpose of obviating the most urgent symptoms. With reference to the use of stimulants in these cases, he might remark that the brain was already excited, and became more so by the administration of alcoholic drinks. We were apt to mistake apparent for absolute weakness, and thus err in the treatment we adopted. In these cases the brain was in an oppressed, and not in an absolutely debilitated condition.

Mr. Crisp read a paper on *croup*, the chief object of which was to recommend that the operation of tracheotomy, in this formidable disease, should be performed as a *dernier resort*. With this view he related several cases, in two or three of which he opened the trachea; but owing to the very late period at which the operation was performed, in consequence of the delay of the parents in giving their consent to the proceeding, no beneficial result followed. In all of these cases, Mr. Crisp believed that if the trachea had been opened when first proposed, life would have been prolonged; and, judging from the morbid appearances in one of the cases, he thought it not improbable that the child might have recovered; the adventitious deposit, in this instance, being confined to the larynx. With the view of placing the operation in question in a more favourable light than it was at present with a majority of the profession, Mr. Crisp referred to a number of cases, in which it had been successfully employed, and directed attention particularly to the cases of Mr. Trousseau, recorded in the "Journal des Connaisances Medico Chirurgicales." In a Table published by this distinguished surgeon, it would appear that he had performed tracheotomy for croup in thirty cases. Of these, eight were successful. In six of the unsuccessful cases three of the patients were dying when the operation was commenced, and they perished in consequence of the inexperience of the assistants. Seven out of the sixteen last operated on were saved. Trousseau observes, that seven of these had been largely bled, and all of them died; thirteen had been moderately depleted, and six of these recovered; four had received no treatment, and two of them did well. With reference to the cause of the disease, Mr. Crisp considers it to depend very frequently on errors in diet; and in regard to the mode in which calomel acts beneficially, he makes the following observations:

"The beneficial effects of calomel appear to to me to arise from its peculiar irritative action upon the mucous lining of the intestines, which is often produced a few hours after its administration. I doubt, however, whether this or any other medicine is likely to be of service in the last stage of the disease."

Dr. Bennett considered that many of the cases of croup related by French practitioners, were different from that inflammatory species of the disease known to English physicians, and that no data could be drawn from them as to the success of tracheotomy. With regard to the operation in cases occurring in this country, he thought we were not justified in having recourse to it early; and that, in the latter stages, in consequence of the implication of the entire respiratory apparatus, it would be useless.—*London Lancet.*

---

*Poisoning by Arsenic.*—M. Orfila detailed to the Royal Academy of Medicine, on the 17th of March, the results of two experiments with arsenic. In the first, he injected twelve grains of arsenious acid into the stomach of a dog. The animal survived an hour and a half; the poison was detected in considerable quantity in the different viscera, but none was found in the urine. In the second experiment, two grains were introduced into the thigh of a dog; the animal died in thirty-six hours, and arsenic was found both in the different viscera and in the urine.—*Ibid.*, *from Archives Generales de Medicine.*

---

*Dumbness produced by Sulphate of Quinine.* By Dr. Ménage.—Madame L., twenty-two years of age, nervous, irregular in menstrual functions, subject to hysterical affections, was seized with intermittent fever, which evinced its activity by the periodic return of the above symptoms. After the six first attacks, twelve grains of sulphate of quinine were ordered, to be exhibited in three doses during the intermission. The two first doses produced no effect; but immediately after taking the third, extreme nervous excitement was induced, the features became sharp, the eyes projecting; there was violent pain in the head, and, finally, a total inability of utterance. The sense of hearing and sight were unaffected. This condition, after lasting for twenty-four hours, ceased instantly, leaving behind it merely a slight confusion in the head. The fever did not reappear.

This case loses much of its interest from occurring in an hysterical patient. A similar case was observed by M. Bertin, and published in a thesis by that gentleman in 1839.—*Lancet, from Gaz. Med. de Paris*, April 25, 1840.

---

*Dumbness produced by Quinine.*—Reading a case of "Dumbness produced by sulphate of quinine," in the last number of the Lancet, related by Dr. Ménage, a similar case, which occurred in my own practice some years since, was brought to my mind. At that time I did not think it worth notice, as I was then doubtful if the sulphate of quinine could cause this affection; but it appears to me now to be of more importance, as, in connection with the cases of Dr. Ménage and M. Bertin, it tends to prove this occasional singular effect.

I was attending Mrs. B., daughter of a retired surgeon, about thirty years of age, (married, and the mother of three children, not subject to hysteria, or any other nervous affection,) with the late Dr. Armstrong, in consequence of intermittent fever, and it was thought right to give her sulphate of quinine. A considerable quantity of that medicine was taken, and I was sent for, in great haste, in consequence of her being incapable of speaking. She was perfectly sensible, with a good countenance, and her respirations were natural. By omitting the quinine she recovered her speech in twelve or fourteen hours. G. Oakley Heming.

*London Lancet.*

---

*Nerves of the Cornea.*—Dr. Pappenheim has succeeded in tracing minute twigs of nerves from the sclerotic coat into the cornea. For this purpose, he immerses the cornea in acetic acid, or in a solution of caustic potass, places it between two plates of glass, and examines it by transmitted light, with a lens of low power. They are most distinctly seen near the periphery of the cornea, where they form plexuses, but become scattered, and appear lost towards the central part. They are smaller than the fibres composing the lamellæ.—*Ibid.*, *from Monatschrift für Medicin*, 1839.

